# Evaluation of the reliability and validity for X16 balance testing scale for the elderly

**DOI:** 10.1186/s12877-018-0803-6

**Published:** 2018-05-10

**Authors:** Jingjuan Ju, Yu Jiang, Peng Zhou, Lin Li, Xiaolei Ye, Hongmei Wu, Bin Shen, Jialei Zhang, Xiaoding He, Chunjin Niu, Qinghua Xia

**Affiliations:** 1 School of Public Health and Management, Wenzhou Medical University, Wenzhou, People’s Republic of China; 2 Changning Center for Disease Control and Prevention, Shanghai, People’s Republic of China

**Keywords:** Elderly, Balance testing scale, Reliability, Validity, Balance

## Abstract

**Background:**

Balance performance is considered as an indicator of functional status in the elderly, a large scale population screening and evaluation in the community context followed by proper interventions would be of great significance at public health level. However, there has been no suitable balance testing scale available for large scale studies in the unique community context of urban China.

**Methods:**

A balance scale named X16 balance testing scale was developed, which was composed of 3 domains and 16 items. A total of 1985 functionally independent and active community-dwelling elderly adults’ balance abilities were tested using the X16 scale. The internal consistency, split-half reliability, content validity, construct validity, discriminant validity of X16 balance testing scale were evaluated.

**Results:**

Factor analysis was performed to identify alternative factor structure. The Eigenvalues of factors 1, 2, and 3 were 8.53, 1.79, and 1.21, respectively, and their cumulative contribution to the total variance reached 72.0%. These 3 factors mainly represented domains static balance, postural stability, and dynamic balance. The Cronbach alpha coefficient for the scale was 0.933. The Spearman correlation coefficients between items and its corresponding domains were ranged from 0.538 to 0.964. The correlation coefficients between each item and its corresponding domain were higher than the coefficients between this item and other domains. With the increase of age, the scores of balance performance, domains static balance, postural stability, and dynamic balance in the elderly declined gradually (*P* < 0.001). With the increase of age, the proportion of the elderly with intact balance performance decreased gradually (*P <* 0.001).

**Conclusions:**

The reliability and validity of the X16 balance testing scale is both adequate and acceptable. Due to its simple and quick use features, it is practical to be used repeatedly and routinely especially in community setting and on large scale screening.

**Electronic supplementary material:**

The online version of this article (10.1186/s12877-018-0803-6) contains supplementary material, which is available to authorized users.

## Background

Aging has been one of the most significant population trends for decades, because the population aged 60 years and over is growing progressively and rapidly in nearly all countries worldwide. In 2012, the population aged 60 years and over represented over 11% of the total global population, and the United Nations Population Fund (UNFPA) estimated that the proportion will be approximately 22% by 2050. In China, there were 180 million people aged 60 years and over in 2012 (UNFPA). By the end of 2015, the population aged 65 years and over accounted for 10.5% of the total population, and this proportion is estimated to increase to nearly one-third in 2050. Shanghai is the most populous city in China. Shanghai has the highest life expectancy in mainland China at 83 years. Additionally, the proportion of the population aged 60 years and older was 28.8% in 2014 [1]. The elderly population is more prone to diseases and disabilities due to declines in their functional, mental, and physical capacities. Therefore, research on the characteristics of aging in the elderly is required.

Balance refers to the ability to maintain the line of gravity of a body within the base of support with minimal postural sway [2]. Functionally, balance may be divided into static balance and dynamic balance. Static balance is the ability to retain the center of mass above the base of support in a stationary position, and dynamic balance is the ability to maintain balance while in motion or switching between positions. The maintenance of balance requires the coordination of the input of multiple systems, including the vestibular, somatosensory, and visual systems [3]. Impaired functions of the above systems may result in a decrease in balance performance, which is associated with the future development or recovery of certain dysfunctions and diseases [4, 5]. Therefore, balance performance is considered an indicator of the functional status in the elderly and has been applied as a measure to quantify functional limitations, determine the need for rehabilitation services, measure clinical changes, and predict health outcomes [6–9].

Functional balance has been frequently evaluated using the Berg Balance Scale, the Timed Up and Go test, and the Performance-Oriented Mobility Assessment (POMA) due to their satisfactory reliability and validity. The Berg Balance Scale is a measure of static and dynamic balance using 14 basic mobility tasks commonly performed in daily life [10–12]. Each item is scored on a scale from 0 to 4 for a maximum of 56 points. This scale takes 15 to 20 min to complete. The Berg Balance Scale is the most commonly used assessment tool for stroke rehabilitation in the clinic and has been frequently applied to identify and evaluate balance impairment in the elderly [13–16].

The POMA provides a dynamic integrated assessment of mobility in the elderly population. The POMA consists of 2 portions that test balance and gait ability during normal daily activities. The balance portion includes 9 maneuvers, and the gait portion includes 7 maneuvers. Each maneuver is scored on a 2- or 3-point ordinal scale with a range of 0 to 1 or 0 to 2 for a maximum total score of 28 points. The POMA requires no equipment and little experience to perform; thus, it can be administered easily in clinical settings. The POMA takes 10 to 15 min to complete [17].

The Timed Get Up and Go test measures dynamic balance and mobility. The subject is observed and timed in seconds while he rises from an arm chair, walks 3 m, turns around, walks back to the chair, and sits down again. The subject starts with his back against the chair, his arms resting on the arms of the chair, and his customary walking aid at hand. The Timed Get Up and Go test is a useful measure of physical mobility for the elderly due to its correlations with extensive measures of balance, gait, and functional abilities. Additionally, the test is easy to perform without requirements for special equipment or training [18].

These above-mentioned tests were originally designed to evaluate balance function in clinical settings. Both static balance and dynamic balance are included in the Berg Balance Scale, although they are not assessed separately. The scoring system of the Berg Balance Scale is subtle, and the differentiation from points 1 to 3 requires careful attention from the investigators. Relative to the Berg Balance Scale, the POMA includes 2 sections for balance and gait that can be scored separately, and its scoring system is simple and easy. The Timed Get Up and Go test quantitatively measures the overall movement ability in seconds, although the specific balance capacity based on the separate tasks is not evaluated.

Due to technological advances, quantitative assessments have also been developed to accurately evaluate the center of pressure [19, 20]. However, instrument-based methods require equipment, technical expertise, personnel, and usually time, which in turn make the assessment subject to variations from various factors.

Balance performance tests have been associated with multiple measures of health status, and the assessments provide valuable information to understand the development of disorders and disabilities. Thus, screening and evaluation in research and geriatric assessment settings may yield a high-risk population with reduced performance whose status can be improved with timely and targeted intervention. Large-scale population screening and evaluation in the community context followed by proper interventions would be of great significance at the public health level. However, there has been no suitable balance testing scale available for large-scale studies in the unique community context of urban China.

Additionally, the duration of testing is a significant concern for large-scale population screening and evaluation in the community population. Because long testing times may increase the chances of participant non-compliance, the expected duration of the testing scale is limited.

Given all these concerns, the X16 balance testing scale for the elderly was developed based on the aforementioned tests. In the X16 scale, the specific balance tasks were rearranged and classified to measure the overall balance performance and individual balance domains simultaneously, and the testing and scoring methods were modified to minimize the expertise requirements for investigators and examiners. The testing duration was limited to 5 min to increase participant compliance. This study aimed to investigate the reliability and validity of the X16 balance testing scale for use in the assessment of balance performance in the elderly.

## Methods

### Subjects and design

The subjects were community-dwelling people randomly selected from Changning District, Shanghai, China. The inclusion criteria included individuals of both genders aged 60 years or older who were able to ambulate without assistance from others or assistive devices, able to understand and answer the interview questions, and able to follow verbal requests for movements. The exclusion criteria included having dementia or visual deficits and being unable to finish the test for other health reasons.

A questionnaire was applied in this study. Data were collected through a face-to-face interview by trained investigators. Balance performance was examined on-site using the X16 balance testing scale for the elderly. The definition of a fall in the present study was an event which results in a person coming to rest on floor or lower level. The history of falls was defined as a fall happened in the past 12 months before the balance test.

The project was approved by Institutional Review Board (IRB) of the Changning Center for Disease Control and Prevention, and written informed consent was obtained from all individual participants included in the study.

### Development of the scale

The X16 Scale was developed based on the principles as below, 1) less than 5 min’ test duration, 2) easy to be mastered by general practitioners from community health centers and can be administered with the minimal need of tools, 3) easy to be understood and followed by the elderly population with low literacy. The main body of the scale was adapted from the BBS and Tinetti POMA scale. Seven items from BBS [12] and 12 from Tinetti POMA scale [17] were selected to form the item pool. Then items were selected based on interview with 6 experts in epidemiology, geriatrics and physical education. The list of items was revised following a pretest of all preliminary items.

The 4 items in domain static balance were mainly derived from BBS, but the scoring was simplified into two categories, one point was scored if the subject could maintain the posture for more than 10 s, zero was scored otherwise. The choices of which leg to stand on or which foot in the front were left to the subject. No assistive device was allowed in this part.

The postural stability domain consists of 4 items, they are 1) Standing to sitting, 2) Sitting to standing, 3) Standing to squatting, and 4) Squatting to standing. The first 2 items were streamlined from both BBS and Tinetti POMA. The last 2 items were added to represent the ability of changing position and finishing tasks such as picking up objects on the floor. Two points were scored for those being able to change position steadily without attempt or help from others, one point for those being able to change position with attempts or assistance, zero point was scored for those not being able to finish the task.

The dynamic balance domain consists of 8 items. Seven items were adapted from the Tinetti POMA and one was from both BBS and Tinetti POMA. The subject was instructed to walk along a 3 m line and turn back to the starting point. The score was given according to the quality of the task. Zero point was scored if the subject initiated the walk after hesitates or attempts, walked with at least 1 foot was unable to lift clearly off the ground, step length was shorter than foot length, step length or step height was non-uniform, walked with stops between steps, walked with deviation from the line, walked with trunk sway or arm extension for balance, or turned with sway or stop.

The X16 scale is composed of 3 domains with a total of 16 items. The full scores for the static balance, postural stability, and dynamic balance domains are 4, 8, and 8 points, respectively; thus, the full score for balance performance is 20 points. The total score for each individual was calculated by summing the scores across the items, and the functional ability was estimated, with higher scores indicating better balance performance. Details are included in Table 1.

**Table 1:**
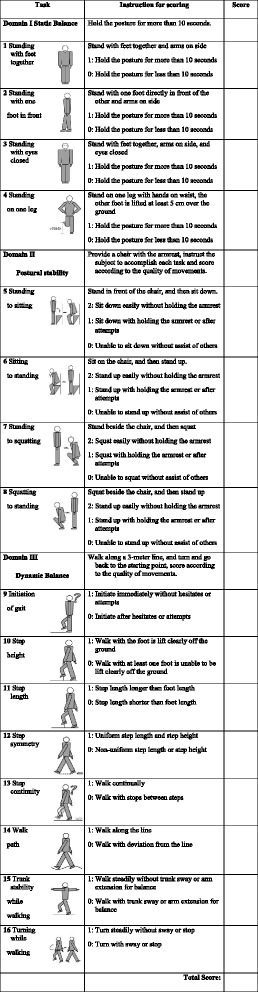
X16 balance testing scale for the elderly

Instruction: the X16 balance testing scale is an objective measure of balance performance, it was designated to identify and evaluate balance impairment in the elderly. There are 16 tasks in the X16 scale, the subject is required to demonstrate each task as instructed and the score is recorded. Make sure that the environment around is safe and subjects are guarded for safety

### Statistical analyses

EpiData 3.0 (The EpiData Association, Odense, Denmark) was used for data entry, and SPSS 18.0 (SPSS Inc. Chicago, IL, USA) was applied for data processing and statistical analysis. The significance level was set at 0.05. Factor analysis and principal components analyses were applied to evaluate hypothesized subscales based on the conceptual framework, and to select the best set of items for this scale. The Cronbach’s alpha coefficient was used for describe the internal consistency. Criteria for internal consistency were Cronbach’s alpha ≥ 0.7 [21]. The construct validity was evaluated by comparing balance ability between age groups in the elderly [22]. The ability of the scale to distinguish between older individuals with or without a history of falls was also tested. Wilcoxon rank-sum test was applied to determine whether a difference of the proportion of balance performance existed between elderly adults with a history of falls and elderly adults without a history of falls. Floor and ceiling effects of this scale was also assessed.

## Results

### Characteristics of the subjects

A total of 1985 participants aged 60 years and over were recruited in this study, among which 940 were men (47.4%), and 1045 were women (52.6%). The median age of the 1985 participants was 69.0 years (range of 60 to 97 years), and the mean ± standard deviation was 70.5 ± 7.5 years.

### The relationship between domains and items

The Kaiser-Meyer-Olkin Measure (KMO) was 0.927, and the Chi-square value of Bartlett’s Test was 24,833.4 (*P* < 0.001). Therefore, a factor analysis was performed to identify alternative factor structures. The Eigenvalues of factors 1, 2, and 3 were 8.53, 1.79, and 1.21, respectively. The variance proportions of factors 1, 2, and 3 were 34.2%, 19.8%, and 18.1%, respectively. Factors 1, 2, and 3 were retained because the Eigenvalues of these 3 factors were greater than 1, and their cumulative contributions to the total variance reached 72.0%. Factor 1 was contributed mainly by the items in the dynamic balance domain, factor 2 was contributed mainly by the items in the postural stability domain, and factor 3 was contributed mainly by the items in the static balance domain [Table 2].

**Table 2:** Factor score coefficient matrix

Domain	Item	Factor		
		1	2	3
I	1	−0.081	− 0.077	**0.395**
	2	−0.055	−0.099	**0.392**
	3	−0.035	−0.070	**0.334**
	4	**−0.136**	**0.160**	**0.216**
II	5	0.029	**0.185**	−0.046
	6	−0.044	**0.288**	−0.047
	7	**−0.152**	**0.432**	−0.047
	8	**−0.169**	**0.455**	−0.056
III	9	**0.229**	**−0.159**	−0.009
	10	**0.199**	−0.076	−0.053
	11	**0.154**	−0.051	−0.029
	12	**0.197**	−0.072	−0.045
	13	**0.164**	−0.032	−0.030
	14	**0.242**	**−0.151**	−0.044
	15	**0.203**	**−0.105**	−0.011
	16	**0.138**	0.012	−0.031

Domain I is static balance, domain II is postural stability, domain III is dynamic balance, and domain IV (sum of domains I, II, and III) is balance performance. Items are named as domain number followed by item number, for example, II 7 indicates the item 7 which is in domain II. Items were numbered consecutively through the whole balance testing scale. The 16 items of the balance testing scale were subjected to factor analysis, the extraction method was principal component analysis, and the rotation method was Varimax with Kaiser Normalization. Three factors were retained because their Eigenvalues were greater than 1 and their cumulative contribution to the total variance reached 72.0%. Numbers with absolute values greater than 0.1 were in bold

The Spearman correlation coefficients between items and their corresponding domains ranged from 0.538 to 0.964. The correlation coefficient between each item and its corresponding domain was greater than the coefficients between this item and the other domains. The correlation coefficients between each item and the total score ranged from 0.428 to 0.790. All *P* values for the Spearman correlation coefficients between each item and domain were less than 0.001 [Table 3].

**Table 3:** Spearman correlation coefficients between items and its corresponding domain and other domains

Domain	Item	Domain			
		I	II	III	IV
I	1	**0.594**	0.234	0.239	0.428
	2	**0.618**	0.280	0.325	0.461
	3	**0.700**	0.365	0.389	0.531
	4	**0.939**	0.452	0.417	0.696
II	5	0.455	**0.712**	0.662	0.623
	6	0.463	**0.772**	0.632	0.662
	7	0.459	**0.923**	0.572	0.770
	8	0.444	**0.964**	0.541	0.790
III	9	0.352	0.432	**0.615**	0.481
	10	0.389	0.502	**0.691**	0.543
	11	0.363	0.457	**0.805**	0.587
	12	0.388	0.530	**0.663**	0.536
	13	0.448	0.554	**0.694**	0.568
	14	0.339	0.435	**0.538**	0.434
	15	0.398	0.503	**0.582**	0.479
	16	0.425	0.587	**0.671**	0.555

Domain I is static balance, domain II is postural stability, domain III is dynamic balance, and domain IV (sum of domains I, II, and III) is balance performance. Items are named as domain number followed by item number, for example, II 7 indicates the item 7 which is in domain II. Items were numbered consecutively through the whole balance testing scale. Numbers in bold are correlation coefficients between each item and its corresponding domain

### Score and categories of balance performance ability

Using hierarchical clustering, the total balance performance scores were classified into 4 categories as follows: category 0 (intact, 17–20 points), category 1 (mildly impaired, 13–16 points), category 2 (moderately impaired, 7–12 points), and category 3 (severely impaired, 0–6 points) balance performance. Category 0 represented the best balance performance, and categories 1 to 3 represented impaired balance performance, with greater numbers indicating more severe impairment.

The floor effects for overall balance performance and the 3 individual domains ranged from 0.7% to 5.5%. The floor effects in the separate age groups ranged from 0.2% to 21.4%. The lowest possible score was 0. With the exceptions of the lowest scores of 4 for overall balance performance and 3 for postural stability in the 60-year-old group, the lowest score for all of the other groups was 0. In the 85–97-year-old group, the ceiling effect for the overall balance performance was 19.4%. The ceiling effect for the overall balance performance and the 3 individual domains was greater than 30% in all other separated and merged age groups [Table 4].

**Table 4:** The floor and ceiling effects of the balance performance and individual domains

Age (years)	*n*	I		II		III		IV	
		Floor effect	Ceiling effect	Floor effect	Ceiling effect	Floor effect	Ceiling effect	Floor effect	Ceiling effect
60-	511	2.5	87.5	0.2	87.5	1.2	87.7	0.4	74.2
65-	530	3.4	82.3	0.4	76.8	0.9	80.8	0.4	60.9
70-	378	3.7	73.3	0.3	71.7	1.1	79.1	0.3	54.2
75-	297	7.4	61.6	1.0	59.3	5.4	69.4	0.3	43.4
80-	166	13.3	51.8	1.8	48.2	11.4	53.0	1.8	34.3
85–97	103	20.4	32.0	7.8	31.1	21.4	35.0	6.8	19.4
Total	1985	5.5	73.7	0.9	71.2	3.6	75.8	0.7	56.1

Domain I is static balance, domain II is postural stability, domain III is dynamic balance, and domain IV (sum of domains I, II, and III) is balance performance. The floor and ceiling effects are in percent

### Reliability

The internal consistency was evaluated. The Cronbach’s alpha coefficient for the balance testing scale was 0.933, and the Cronbach’s alpha coefficients for the static balance, postural stability, and dynamic balance domains ranged from 0.823 to 0.923. The Spearman correlation coefficients between the individual domains and balance performance were ranged from 0.742 to 0.831, which were higher than the correlation coefficients between individual domains (from 0.460 to 0.618). All *P* values for the Spearman correlation coefficients were less than 0.001 [Table 5].

**Table 5:** Cronbach alpha coefficients and Spearman correlation coefficients

Domain	I	II	III	IV
I	**0.823**			
II	0.481	**0.915**		
III	0.460	0.618	**0.923**	
IV	0.742	0.831	0.747	**0.933**

Domain I is static balance, domain II is postural stability, domain III is dynamic balance, and domain IV (sum of domains I, II, and III) is balance performance. Numbers in bold are Cronbach alpha coefficients, others are Spearman correlation coefficients

The split-half reliability of the balance testing scale and the three individual domains were assessed with the Spearman-Brown coefficients, Guttman split-half coefficients, and Hotelling’s T-Squared test. The Spearman-Brown and Guttman split-half coefficients ranged from 0.844 to 0.968. All of the coefficients for the scale were higher than the coefficients for the domains (Additional file 1: Table S1).

### Validity

The construct validity was evaluated. Differences in the balance performance scores between age groups in the elderly were analyzed with one-way analysis of variance (ANOVA), followed by Tamhane’s T2 for multiple comparisons. The balance performance scores in the elderly declined with increasing age. The balance performance score in 60–70-year-old group was significantly higher than the score in the 70–80-year-old and 80–97-year-old groups (all *P* < 0.001), and the score in 70–80-year-old group was significantly higher than the score in the 80–97-year-old group (*P* < 0.001). Similar patterns were found for the scores of the static balance, postural stability, and dynamic balance domains (all *P* < 0.001). The data are shown in Table 6.

**Table 6:** Age-specific balance performance of the elderly in Shanghai

Age (years)	*n*	Static balance	Postural stability	Dynamic balance	Balance performance
60~	1041	3.72 ± 0.82^a^	7.56 ± 1.10^a^	7.63 ± 1.20^a^	18.91 ± 2.47^a^
70~	675	3.41 ± 1.08^b^	7.01 ± 1.62^b^	7.19 ± 1.87^b^	17.61 ± 3.84^b^
80~ 97	269	2.73 ± 1.47^c^	5.77 ± 2.30^c^	5.62 ± 3.07^c^	14.12 ± 6.06^c^
Total	1985	3.48 ± 1.07	7.13 ± 1.61	7.21 ± 1.91	17.82 ± 3.96

The data were presented as mean ± standard deviation. The one-way ANOVA was performed followed by Tamhane’ T2 for multiple comparison

Letters (a, b, c) indicated the multiple comparison results among various ages. Same letters indicated non-significant difference, different letters indicated significant differences in statistics. Significance level was 0.05

The proportions of balance performance in the elderly at various ages were compared with Pearson’s Chi-square test. The proportion of the elderly with intact balance performance decreased gradually with increasing age (*P <* 0.001). At 60–70 years of age, 88.4% of the elderly had intact balance performance; this proportion decreased to 74.5% at 70–80 years of age and substantially decreased to 46.8% after 80 years of age [Table 7].

**Table 7:** Age-specific proportions of balance performance in the elderly in Shanghai

Age (years)	Subtotal		0		1		2		3	
	*n*	%	*n*	%	*n*	%	*n*	%	*n*	%
60~	1041	52.4	920	88.4	90	8.6	20	1.9	11	1.1
70~	675	34.0	503	74.5	108	16.0	43	6.4	21	3.1
80~ 97	269	13.6	126	46.8	57	21.2	42	15.6	44	16.4
Total	1985	100.0	1549	78.0	255	12.9	105	5.3	76	3.8

Numbers 0, 1, 2, and 3 indicate the categories of balance performance, they are category 0 (intact), category 1 (mildly impaired), category 2 (moderately impaired), and category 3 (severely impaired) balance performance. Pearson Chi-Square value is 293.1, *P <* 0.001

Balance performance was compared between the elderly with and without a history of a fall in the past 12 months (Table 8). The fall rate of the 1985 participants was 17.3%. There was a difference in balance performance between the fall and non-fall groups (Wilcoxon rank-sum test, z = 5.579, *P* < 0.001). The proportion of intact balance performance in the non-fall group was significantly higher than the proportion in the fall group (Chi-square value = 24.788, *P* < 0.001). The logistic regression analysis results showed that the impairment in balance performance was potentially associated with an increased risk of falls, with OR = 1.585 (95% CI, 1.378–1.823, *P* < 0.001).

**Table 8:** Relationships between balance performance and fall in the elderly

Fall	n	0	1	2	3
Yes	343	220 (64.1)	65 (19.0)	34 (9.9)	24 (7.0)
No	1642	1264 (77.0)	272 (16.6)	73 (4.4)	33 (2.0)
Total	1985	1484 (74.8)	337 (17.0)	107(5.4)	57 (2.9)

Values were presented as number (percent). Numbers 0, 1, 2, and 3 indicate the categories of balance performance, they are category 0 (intact), category 1 (mildly impaired), category 2 (moderately impaired), and category 3 (severely impaired) balance performance

## Discussion

In this study, the reliability and validity of the X16 balance testing scale were evaluated for its use in community-dwelling elderly. The results demonstrated that the use of the scale was both adequate and acceptable.

Factor analysis reached a three-factor solution. Factor 1 was contributed mainly by the 8 items in the dynamic balance domain, 2 items from the postural stability domain, and 1 item from the static balance domain. Factor 2 was contributed mainly by the 4 items in the postural stability domain, 1 item from the static balance domain, and 3 items in the dynamic balance domain. Factor 3 was contributed mainly by the 4 items in the static balance domain. These results showed that some items contributed to multiple factors; therefore, the contributions of each item across factors were compared, and the major contribution of each item was determined. The results demonstrated that these 3 factors primarily represented the static balance, postural stability, and dynamic balance domains, respectively. Thus, the results proved that X16 scale had sound reason to be divided into 3 domains.

The correlation coefficients were highest between each item and its own domain, followed by the correlations between each item and the entire scale. The correlations were lowest between each item and the other domains. These results further confirmed the structure of the X16 scale was well designed [23].

All of the floor effects for the X16 scale and the individual domains in all age groups were less than 30%, indicating that their floor effects were acceptable. The ceiling effects for the X16 scale and the individual domains were greater than 30%. The aim of this study was to evaluate the functional balance of the elderly and to identify individuals with impaired balance for further intervention at the individual and public health levels. The scoring system is mostly based on whether an individual person can function independently in terms of mobility. Thus, the highest possible score indicates that the individual person is active and independent in their movements in daily life but does not indicate the perfectness of the balance function. Therefore, the ceiling effects were relatively high. It varied in different age groups, which suggested that the scale might be more useful in people over 70.

Reliability is the overall consistency of a measurement, and the estimation of reliability provides information about the amount of random error from the measurement process [24]. The internal consistency reliability was determined for the X16 scale and its 3 domains. The Cronbach’s alpha coefficients were all greater than 0.7, indicating achievements of excellent internal consistency for the X16 scale and each domain.

All of the correlations between each item and the domains were significant. These results suggested that the X16 scale possessed effective reliability.

The validity of an assessment is the degree to which evidence and theory support the interpretations of the test scores [24]. The construct validity was evaluated for the X16 scale.

The balance performance of the elderly was compared between age groups. The subjects with older ages had lower scores for overall balance performance and the separate domains. Additionally, the proportions of the elderly with intact balance performance decreased gradually with increasing age, whereas the proportions of the elderly with impaired balance increased stably and substantially with increasing age. These findings indicate that the X16 scale and the domains possess discriminative abilities to differentiate the functional statuses of populations with different ages.

The balance performance was further compared between the elderly without and with a history of a fall in the past 12 months. The average balance performance of the elderly without a fall history was better than the performance of the elderly with a fall history; the elderly without a fall history had a higher proportion of intact balance performance, whereas impairment in balance performance increased the fall risk. These analytical results indicate that the balance performance measured by the X16 scale was associated with balance-related disorders and disabilities.

A valid and quantitative measure of senior balance is of significance for both practice and research. The X16 scale was developed to evaluate the balance function with quantitative measures of overall balance performance and individual balance domains in the elderly. This scale is simple and applicable without special requirements for expertise or training, and it takes only 3 to 5 min to administer. It is potential to be used in assessing the balance capacity and screening people at high risk of falling then taking measures to prevent falls at an earlier stage. The present study made exploring analyses regarding reliability and validity. However, there are limitations in this study. First, the evidence of validity and reliability are specific to urban community of China and Chinese elderly adults. Second, we didn’t compare the X16 scale to fine validated balance assessment methods such as the Berg Balance Scale, POMA, or the Short Physical Performance Battery. Third, due to the cross-sectional design, we couldn’t provide predictive values in this study. To further verify the scale as a potential screening test, applying in different settings or populations, comparing to golden standards, and conducting prospective studies will allow the evaluation of sensitivity, specificity, predictive values, false results, and the sample size needed to screen to prevent one fall.

## Conclusions

The reliability and validity of the X16 balance testing scale is both adequate and acceptable. It is potential to be used in assessing the balance capacity and screening people at high risk of falling then taking measures to prevent falls at an earlier stage. Due to its simple and quick use features, it is practical to be used repeatedly and routinely especially in community setting and on large scale screening.

## Additional file

Additional file 1: **Table S1.** Split-Half coefficients. (DOCX 15 kb)

## Abbreviations

ANOVA: Analysis of variance
IRB: Institutional Review Board
POMA: Performance-Oriented Mobility Assessment
UNFPA: The United Nations Population Fund
