# Supplementary material for: Evaluation of the reliability and validity for X16 balance testing scale for the elderly
Source: BMC Geriatr. 2018 May 10;18:112. doi: 10.1186/s12877-018-0803-6 (PMC5971429; doi:10.1186/s12877-018-0803-6)
Supplement: Supplementary file 1 — Table S1. Split-Half coefficients. (DOCX 15 kb) [file 12877_2018_803_MOESM1_ESM.docx]

**Table S1 Split-Half coefficients**

| Domain | Correlation between forms | Spearman-  Brown coefficient | Guttman Split-Half coefficients | Hotelling's T-Squared test | |
| --- | --- | --- | --- | --- | --- |
|  |  |  |  |  |  |
|  |  |  |  | F | *P* |
| I | 0.732 | 0.846 | 0.844 | 115.0 | <0.001 |
| II | 0.918 | 0.957 | 0.957 | 112.5 | <0.001 |
| III | 0.883 | 0.938 | 0.937 | 30.3 | <0.001 |
| IV | 0.938 | 0.968 | 0.968 | 2664.9 | <0.001 |

Domain I is static balance, domain II is postural stability, domain III is dynamic balance, and domain IV (sum of domains I, II, and III) is balance performance. Items are named as domain number followed by item number, for example, II 7 indicates the item 7 which is in domain II. Items were numbered consecutively through the whole balance testing scale. Items in each domain were split in half. Items for subscale 1 are I 1 and I 4, II 5 and II 8, III 9, III 11, III 13, and III 15. Items for subscale 2 are I 2 and I 3, II 6 and II 7, III 10, III 12, III 14, and III 16.
